# Engineering large, anatomically shaped osteochondral constructs with robust interfacial shear properties

**DOI:** 10.1038/s41536-021-00152-0

**Published:** 2021-08-06

**Authors:** Wendy E. Brown, Brian J. Huang, Jerry C. Hu, Kyriacos A. Athanasiou

**Affiliations:** 1 Department of Biomedical Engineering, University of California Irvine, 3120 Natural Sciences II, Irvine, CA USA; 2 Integrative Stem Cell Center, China Medical University Hospital, Taichung, Taiwan

**Keywords:** Tissue engineering, Tissues, Regenerative medicine

## Abstract

Despite the prevalence of large (>5 cm^2^) articular cartilage defects involving underlying bone, current tissue-engineered therapies only address small defects. Tissue-engineered, anatomically shaped, native-like implants may address the need for off-the-shelf, tissue-repairing therapies for large cartilage lesions. This study fabricated an osteochondral construct of translationally relevant geometry with robust functional properties. Scaffold-free, self-assembled neocartilage served as the chondral phase, and porous hydroxyapatite served as the osseous phase of the osteochondral constructs. Constructs in the shape and size of an ovine femoral condyle (31 × 14 mm) were assembled at day 4 (early) or day 10 (late) of neocartilage maturation. Early osteochondral assembly increased the interfacial interdigitation depth by 244%, interdigitation frequency by 438%, interfacial shear modulus by 243-fold, and ultimate interfacial shear strength by 4.9-fold, compared to late assembly. Toward the development of a bioprosthesis for the repair of cartilage lesions encompassing up to an entire condylar surface, this study generated a large, anatomically shaped osteochondral construct with robust interfacial mechanical properties and native-like neocartilage interdigitation.

## Introduction

Injuries resulting in articular cartilage defects represent a substantial burden to the U.S. and international healthcare systems. Cartilage does not heal because it is avascular and has low cellularity. Small cartilage lesions progressively deteriorate to form large defects, causing great physical pain. While recent clinical trends aim to repair cartilage defects when they are small, many people are still afflicted with large articular cartilage lesions. In a study of cadaveric human knees with at least one cartilage lesion, ~40% of defects were characterized as large, covering 15–23% of the femoral condyle and patellofemoral groove^1^, which corresponds to a surface area of 12–18 cm^2^ ^2^. Large defects, if left untreated, continue to degenerate toward osteoarthritis. Approximately 30.8 million people in the U.S., or 13.4% of the adult population, experience osteoarthritic degeneration of their articular cartilage^3^. The worldwide economic burden of osteoarthritis is $89 billion^4^. Large defects represent a high percentage of observed articular cartilage defects, and, thus, a main source of disability. The creation of a unicompartmental bioprosthesis (i.e., an all-biological prosthesis capable of replacing one entire compartment of the knee) would address a substantial unmet clinical need and may obviate the use of synthetic prosthetics.

Current repair therapies are primarily indicated for small focal defects without addressing large (>5 cm^2^) defects^5^. For example, microfracture, recommended for focal lesions of 2–2.5 cm^2^ ^6,7^_,_ cannot treat large defects because sufficient peripheral native cartilage is needed for mechanical support of the marrow clot^8^. Furthermore, with this method, the repair fibrocartilage that is formed is mechanically inferior to native cartilage and deteriorates after 18 months post-surgery^9^. The use of osteochondral autografts is also limited to repairing small defects (<2 cm^2^) because healthy graft tissue, whose isolation causes donor site morbidity, is scarce within an already damaged joint^5^. Furthermore, using this technique to repair large defects is difficult because of the need to match the contours of the articular surface. Osteochondral allografts are used to treat defects of 4 cm^2^ on average^6,7^, but graft availability is limited^10^. The functional properties of osteochondral grafts also decrease in storage, resulting in a limited shelf life^11^. Matrix-induced autologous chondrocyte implantation (MACI) has been used to treat lesions up to 20 cm^2^ but is most frequently used to treat defects <5 cm^2^ ^12^. However, clinical outcomes after MACI plateau at five years and deteriorate by 10 years post-surgery^13^. The only treatments currently indicated for large articular cartilage defects are unicompartmental knee arthroplasty (UKA) and total knee arthroplasty (TKA). UKA is preferred to TKA because it preserves the normal ligamentous structures and kinematics of the knee, leading to faster patient rehabilitation^14^. However, when UKA fails, TKA is required, and the conversion from UKA to TKA is associated with poorer clinical outcomes than primary TKA^15^. Neither UKA nor TKA is tissue-sparing; both represent end-stage therapies that have limited lifespans and require progressively invasive revisions. These shortcomings make UKA and TKA unsuitable for young patients. Because no reparative therapies are indicated for the treatment of large articular cartilage defects, affected patients face either temporary, palliative treatments or invasive replacement of damaged tissues with synthetic implants.

Producing native-like cartilage implants via tissue engineering has the potential to effectively regenerate damaged cartilage and halt the degenerative cascade. The formation of small, engineered neocartilage constructs with native-like organization, composition, and mechanics has been reported^16,17^. For example, articular cartilage zones have been mimicked with the use of poly(ethylene glycol) and chondroitin sulfate hydrogels in a gradient of stiffness and poly-(ε-caprolactone) scaffolds with aligned, fibrous layers^16,17^. The self-assembling process has been shown to generate scaffold-free neocartilage, derived from passaged chondrocytes of various cartilage sources, with functional properties on par with native cartilage values^11,18–20^. Tissue engineering has made strides toward addressing the scarcity of graft tissue and the inferior quality of repair cartilage associated with current treatments. However, engineering large tissues of anatomical size and shape must additionally be achieved.

The main challenges to tissue-engineering robust neocartilage of clinically relevant sizes and shapes are maintenance of construct shape and achieving adequate nutrient diffusion to support the development of biomimetic neocartilage functional properties. For example, hemispherical constructs in the shape and size of a juvenile minipig femoral head (35 mm-diameter) were created by casting methacrylate-modified hyaluronic acid hydrogels containing juvenile bovine mesenchymal stem cells (MSCs) in a 3D printed mold^21^. At the end of a 12-week culture period, the constructs maintained their hemispherical shape. However, reduced extracellular matrix (ECM) content was observed in the center of the construct. In an effort to overcome nutrient diffusion limitations in large contiguous constructs, a separate study assembled small, puzzle piece-shaped agarose blocks containing P2 juvenile bovine chondrocytes into a flat, 13 × 13 mm square. The small puzzle pieces and, thus, the resulting large construct composed of interlocking puzzle pieces had significantly greater compressive equilibrium Young’s modulus, glycosaminoglycan (GAG) content, and collagen content than those properties of a large contiguous construct^22^. However, the interfaces between the interlocking puzzle piece constructs were weak, leading to constructing failure after 49 weeks of culture. Computational models have also been used to predict glucose requirements and to develop large neocartilage engineering strategies^23^. The inclusion of nutrient channels within 10 mm-diameter, flat, cylindrical, agarose constructs containing primary juvenile bovine chondrocytes increased GAG/wet weight content and collagen/wet weight content by 2.6 times and 2.2 times, respectively over constructs that did not contain nutrient channels^23^. Alternative to the use of scaffolds, the self-assembling process has been used to generate flat, cylindrical, scaffold-free neocartilage constructs of 9.23 cm^2^ (~35 mm-diameter) from P4 fetal ovine chondrocytes without a reduction in functional properties compared to 5 mm-diameter constructs^24^. The application of mechanical loads and a chemical treatment regimen consisting of cytochalasin D (cyto D), TGF-β1, chondroitinase ABC (c-ABC), and lysyl oxidase-like 2 (LOXL2) was critical to allow such a scale-up. The self-assembling process holds promise to alleviate the limitations of current cartilage repair therapies, namely those associated with size, tissue scarcity, and inadequate mechanics, by creating anatomically shaped, mechanically functional neocartilage from passaged chondrocytes.

Because large cartilage lesions often involve the underlying bone, the restoration of the integrity and function of not only cartilage but also of subchondral bone is crucial for the regeneration of articular cartilage. Restoration of the subchondral bone architecture has been shown to be important for lasting repair of full-thickness cartilage defects^25^. Bone-cartilage crosstalk also plays an important role in cartilage homeostasis and the pathogenesis of osteoarthritis^26,27^. In addition, bone-to-bone interfaces heal more quickly and completely than cartilage-to-cartilage interfaces^28^. Therefore, the osseous phase of a tissue-engineered osteochondral construct may serve to anchor the implant in the defect^29,30^. This is particularly necessary for large implants because they may be more susceptible to mechanical forces within the joint that causes implant dislocation. This anchoring strategy may also eliminate the use of tissue-damaging sutures and pins or the use of glues that create a barrier to chondral integration. Thus, osteochondral strategies to engineer clinically sized cartilage repair therapies provide several advantages over cartilage-only strategies.

Flat, small (≤5 mm-diameter) osteochondral constructs have been fabricated using various strategies. Strategies include seeding chondrocytes on top of porous calcium phosphate-composite scaffolds^29,31^ or juxtaposing a chondral phase with an osseous phase, such as through suturing^30^. However, the formation of curved shapes or a thick chondral phase is difficult to achieve with direct seeding because the placement of chondrocytes is largely dictated by gravity. Direct suturing or other external methods of anchoring the chondral and osseous phases together allow for the generation of shape-specific constructs but may result in damage to the engineered tissues and incomplete contact between the phases, resulting in poor interfacial mechanics. Small, flat osteochondral constructs have been generated from self-assembled neocartilage and porous hydroxyapatite (HAp) with a consistent and well-adhered osteochondral interface^32^. Importantly, the chondral phase of these constructs maintained compressive and tensile properties on par with control neocartilage that was not exposed to HAp^32^. Osteochondral assembly during the transition from the production of immature cartilage matrix components to matrix maturation allowed for neocartilage interdigitation into the HAp without disruption of the self-assembly process^33,34^. Therefore, the timing of osteochondral assembly is critical in achieving a strong osteochondral interface and neocartilage functional properties. While significant strides have been made toward the fabrication of engineered osteochondral constructs, shortcomings, such as overall construct size and shape, as well as incomplete contact between the phases leading to poor interfacial properties, need to be addressed.

Toward the development of a unicompartmental bioprosthesis to treat large articular cartilage defects, the objectives of this study were to (1) fabricate an osteochondral construct in a translationally relevant size and shape, (2) engineer an interdigitated, robust interface between the chondral and osseous phases of the construct, and (3) determine whether the scale-up process to engineer a large neocartilage phase or exposure to HAp are detrimental to neocartilage mechanical properties. These objectives were approached using a translationally relevant in vitro sheep model. Passage 4 (P4), redifferentiated juvenile ovine articular chondrocytes (joACs) were used to generate self-assembled neocartilage constructs.

The first objective was to fabricate an osteochondral construct in a translationally relevant size and shape. Toward this, custom and shape-specific 3D-printed neocartilage self-assembly molds were generated based on 3D scans of the distal femoral condyle of sheep. HAp ceramics with 55% porosity and compressive strength of 0.95 MPa^32^ were also machined to anatomical shapes based on the 3D scans. Osteochondral constructs were evaluated grossly at the end of culture for the maintenance of fidelity of the anatomical size and shape.

The second objective was to engineer an interdigitated, robust interface between the chondral and osseous phases of the construct. Toward this, the osseous HAp phase was introduced to the self-assembled neocartilage phase at two neocartilage maturation stages to determine the degree of neocartilage maturation necessary for robust osteochondral assembly: day 4 (Early osteochondral [OC] Assembly) or day 10 (Late OC assembly). At day 4, self-assembled neocartilage is in cell interaction and matrix production phase (phase 3)^35^, demonstrated by a peak in N-cadherin^34^ and the presence of immature collagen precursors^33^. At day 10, self-assembled neocartilage is in a maturation phase (phase 4)^35^, when crosslinking of existing collagen occurs. To evaluate the integrity of the osteochondral interfaces generated at each time point, histology of the interface, quantification of the neocartilage interdigitation depth and frequency, and measurement of the interfacial shear properties were performed.

**Fig. 1:**
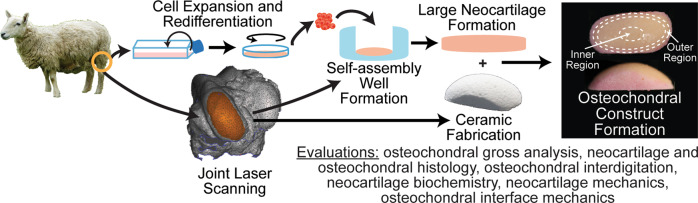
Experimental overview. 3D scans of the medial femoral condyle of juvenile sheep were used to generate large, anatomically shaped neocartilage self-assembly wells and hydroxyapatite ceramics. Passage 4, redifferentiated juvenile ovine articular chondrocytes were used to generate scaffold-free neocartilage. At days 4 or 10 of neocartilage maturation, the neocartilage and ceramics were assembled to form osteochondral constructs. All evaluations took place at the end of the 35-day culture period.

Toward the third objective of evaluating if there were deleterious effects to neocartilage mechanical properties from size scale-up or HAp exposure, the inner (center) and outer (peripheral edge) regions (Fig. 1) of the large neocartilage chondral phase were compared biochemically and mechanically. In addition, because of cartilage’s primary role as a mechanically capable tissue, the mechanical properties of the large neocartilage chondral phase were compared to those of the small, control neocartilage constructs.

## Results

### Overview

Osteochondral constructs were formed (Fig. 1) by assembling large, anatomically shaped HAp ceramics with large, scaffold-free neocartilage constructs at days 4 or 10 of neocartilage maturation. Osteochondral constructs were lightly compressed with static weights to encourage full contact of the chondral and osseous phases. Also applied were cytochalasin D (cyto D), TGF-β1, chondroitinase-ABC (c-ABC), and lysyl oxidase-like 2 protein to enhance the neocartilage functional properties. Small control neocartilage (SCN) constructs were also self-assembled from the same cell source and underwent the same chemical stimulation regimen as the large neocartilage phase of the osteochondral constructs. The success of achieving the objectives was evaluated based on osteochondral gross morphology, osteochondral and neocartilage histology, neocartilage biochemistry, osteochondral interfacial mechanics, and neocartilage mechanics.

### Osteochondral assembly timing shows gross morphological differences

**Fig. 2:**
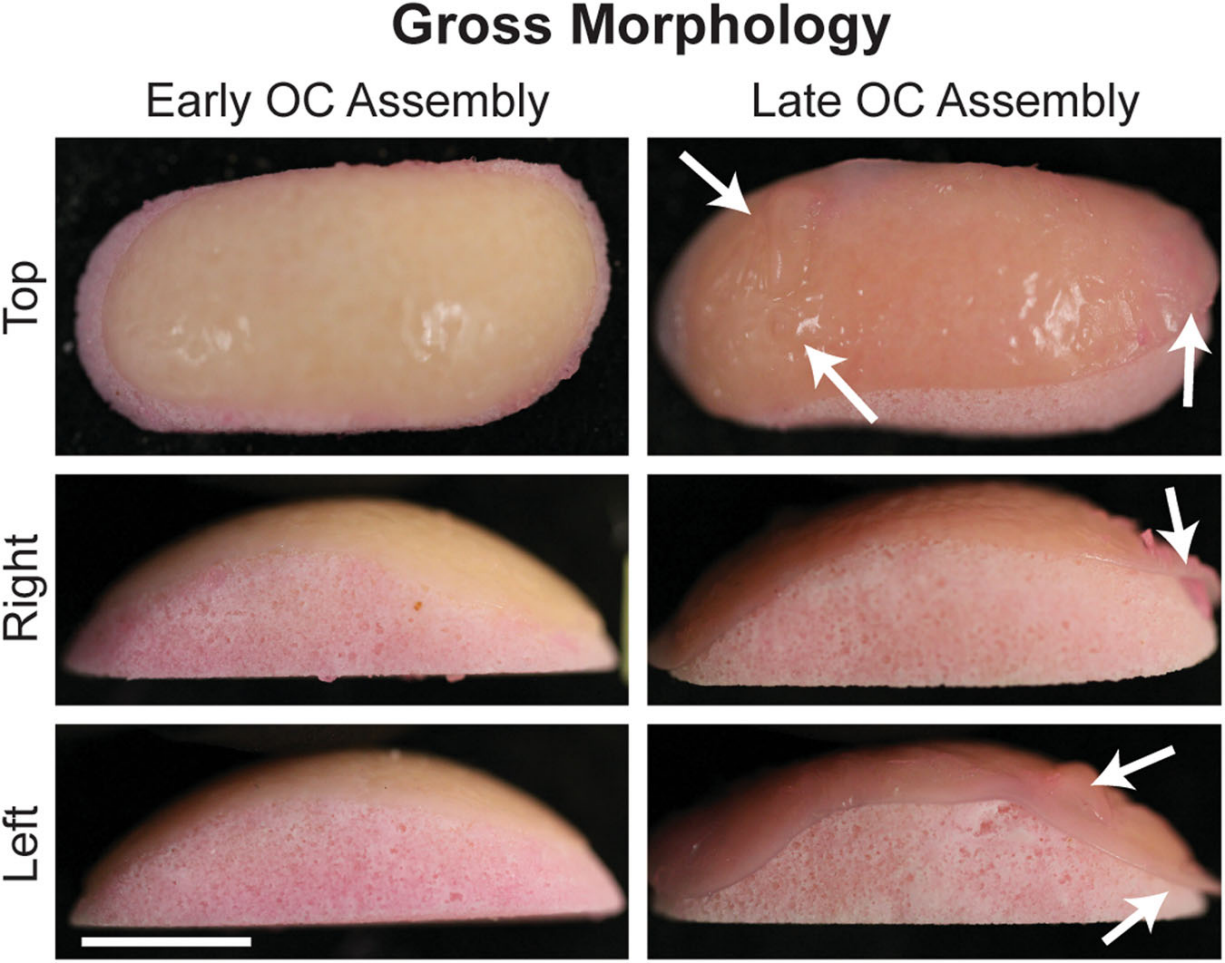
Construct gross morphology. Top, right, and left views of osteochondral construct gross morphology. Arrows indicate regions where the neocartilage was deformed and not in contact with the HAp ceramic. The scale bar represents 10 mm. OC osteochondral.

Prominent morphological differences existed between the Early and Late OC Assembly groups (Fig. 2). In the Early OC Assembly group, the neocartilage appeared homogeneous, smooth, flat, and entirely adhered to the ceramic surface. In the Late OC Assembly group, the neocartilage showed surface irregularities, pockets where it was not adhered to the ceramic and lifted edges.

### Histology shows matrix-rich neocartilage and native-like osteochondral interdigitation in the Early OC Assembly group

**Fig. 3:**
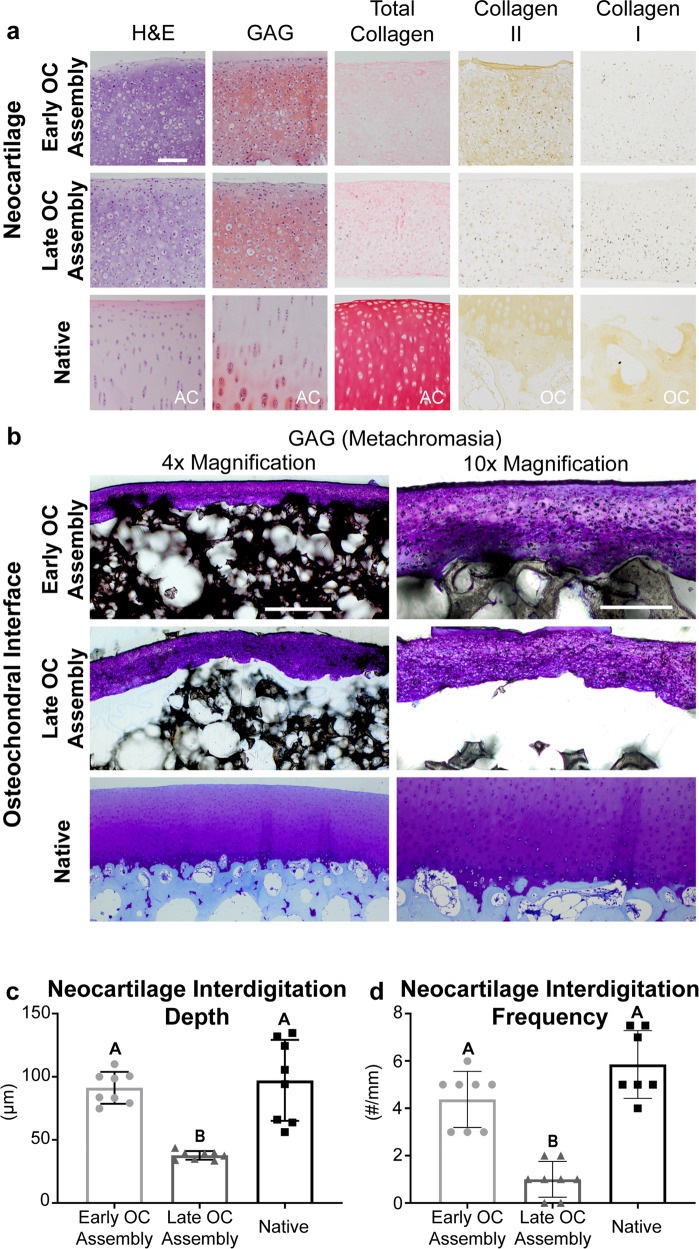
Construct histology and immunohistochemistry. **a** Representative images of histological and immunohistochemical staining of the neocartilage phase of the osteochondral constructs from both groups as well as juvenile ovine articular cartilage (AC) or decalcified osteochondral (OC) tissue controls. The superficial, free-facing surface of the neocartilage is positioned at the top, with the interfacial side at the bottom in each image. Articular cartilage is rich in collagen II and devoid of collagen I, and therefore serves as a positive control for collagen II staining and negative control for collagen I staining. Alternatively, the subchondral bone is rich in collagen I and devoid of collagen II, thus serving as a positive control for collagen I staining and negative control for collagen II stainings. The scale bar represents 100 μm. **b** Toluidine blue staining of plastic-embedded sections and native ovine osteochondral tissue controls. The scale bar represents 500 μm in the 4× images and 200 μm in the 10× images. **c** Comparison of interdigitation depth in both osteochondral assembly groups and native osteochondral tissue, assessed by one-way analysis of variance (ANOVA). **d** Comparison of interdigitation frequency in both osteochondral assembly groups and native osteochondral tissue, assessed by Kruskal–Wallis test due to non-normal distribution of the data. Data are presented as means with errors bars representing standard deviations. H&E hematoxylin and eosin, GAG glycosaminoglycans, OC osteochondral, AC articular cartilage.

Neocartilage from both osteochondral assembly groups was removed from the ceramic and evaluated histologically (Fig. 3a). Neocartilage from both the Early and Late OC Assembly groups showed a higher cellular density compared to native juvenile sheep articular cartilage. Both groups had comparable GAG staining to native cartilage. Collagen staining in the neocartilage of the Early OC Assembly group appeared to be more localized to the superficial and deep (proximal to the HAp) zones, similar to the staining pattern in native juvenile sheep articular cartilage. Neocartilage of the Late OC Assembly group was stained homogeneously throughout the section with comparable intensity to the Early OC Assembly group. Both groups stained less intensely than native articular cartilage. No mineralization via von Kossa and alizarin red staining was observed in either OC assembly group (Supplementary Fig. 1). Neocartilage from the Early OC Assembly group was stained to a similar degree as native juvenile sheep articular cartilage for collagen II and much more intensely than the Late OC Assembly group. Minimal collagen I staining was observed in both groups.

Plastic-embedded sections stained with toluidine blue showed the interface between the chondral and osseous phases in both osteochondral assembly groups, as well as the cartilage and subchondral bone in native ovine osteochondral tissue (Fig. 3b). Neocartilage from the Early OC Assembly group exhibited consistent interdigitation into the osseous phase. The Late OC Assembly group showed large gaps between the chondral and osseous phases. Quantification of histological sections revealed that neocartilage interdigitation depth in the Early OC Assembly group significantly (*p* < 0.0001) exceeded that of the Late OC Assembly group and was on par with that of native sheep osteochondral tissue (Fig. 3c). Similarly, interdigitation frequency in the Early OC Assembly group significantly exceeded (*p* = 0.01) that of the Late OC Assembly group and was on par with that of native sheep osteochondral tissue (Fig. 3d).

### Neocartilage biochemical analysis shows regional homogeneity in the Early OC Assembly group

Neocartilage from both osteochondral assembly groups was removed from the ceramic and analyzed for biochemical content. Neocartilage from the outer and inner regions of the neocartilage within each OC Assembly group was compared to examine tissue homogeneity and potential limitations in nutrient diffusion. Within the Early OC Assembly group, no differences in biochemical content were observed between the outer and inner regions of the neocartilage (Table 1). Within the Late OC Assembly group, GAG/DNA, collagen/wet weight (WW), collagen/dry weight (DW), and collagen/DNA contents were significantly greater (*p* = 0.0051, 0.0150, 0.0051, and 0.0076, respectively) in the outer region of the neocartilage than in the inner region (Table 1).

**Table 1:** Neocartilage regional biochemical content.

Group	Region	Hydration [%]	GAG/WW [%]	GAG/DW [%]	GAG/DNA [μg/μg]	Col/WW [%]	Col/DW [%]	Col/DNA [μg/μg]
Early OC Assembly	Outer	85.6 ± 2.0	1.7 ± 0.4	11.4 ± 1.5	80.4 ± 13.2	1.0 ± 0.1	7.2 ± 0.8	8.0 ± 1.4
	Inner	86.3 ± 0.6	1.3 ± 0.3	9.8 ± 1.7	75.0 ± 12.7	0.9 ± 0.1	6.5 ± 0.6	7.5 ± 1.4
Late OC Assembly	Outer	86.7 ± 1.2	1.5 ± 0.8	10.6 ± 5.7	114.9 ± 25.6^a^	1.1 ± 0.2^a^	8.4 ± 1.2^a^	11.5 ± 2.6^a^
	Inner	87.8 ± 1.0	0.9 ± 0.2	8.2 ± 2.2	82.1 ± 12.1^b^	0.8 ± 0.1^b^	6.8 ± 0.4^b^	8.2 ± 1.2^b^

Biochemical content was compared by region of neocartilage within each OC Assembly group. Paired *t*-tests comparing the inner vs. outer regions within the Early and Late OC Assembly groups were performed.

*GAG* glycosaminoglycans, *Col* collagen, *WW* wet weight, *DW* dry weight, *OC* osteochondral.

Data are presented as means ± standard deviations. Groups marked by different letters (i.e., a and b) are statistically different.

**Fig. 4:**
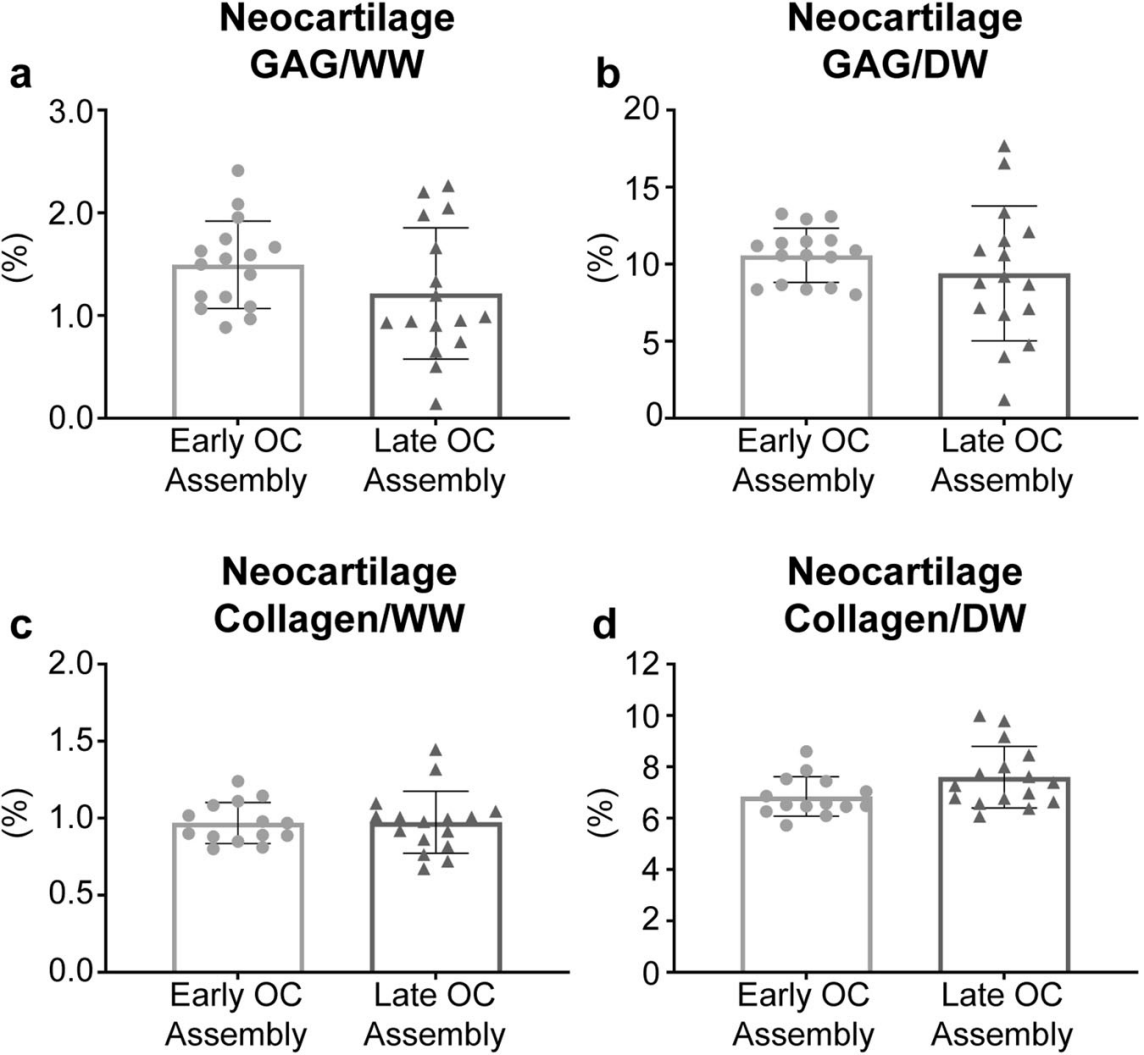
Neocartilage biochemical properties at the end of 5 weeks of culture. **a** GAG/wet weight (WW), (**b**) GAG/dry weight (DW), (**c**) collagen/WW, (**d**) collagen/DW contents are shown. Data are presented as means with errors bars representing standard deviations. Data were analyzed with a student’s *t*-test, and no statistical differences between groups were detected. GAG glycosaminoglycans, WW wet weight, DW dry weight, OC osteochondral.

There were no differences between the Early and Late OC Assembly groups with respect to neocartilage GAG/WW, GAG/DW, collagen/WW, or collagen/DW contents (Fig. 4). GAG/DNA content in the neocartilage from the Late OC Assembly group (101.7 ± 18.1 µg/µg) was significantly greater (*p* = 0.0099) than in the Early OC Assembly group (78.5 ± 9.2 µg/µg). Neocartilage collagen/DNA content in the Late OC Assembly group (10.2 ± 1.8 µg/µg) was significantly greater (*p* = 0.0099) than in the Early OC Assembly group (7.8 ± 0.9 µg/µg).

### The mechanical evaluation demonstrates robust neocartilage in both groups, but a significantly enhanced osteochondral interface in the early OC assembly group

Osteochondral samples from both OC assembly groups were tested in compression and shear. Neocartilage was also removed from the ceramics in both groups and tested in tension. Within the Early OC Assembly group, there were no differences in aggregate modulus, permeability, tensile modulus, or tensile strength between the inner and outer regions of the constructs (Table 2). Within the Early OC Assembly group, the shear modulus of the outer region was significantly greater (*p* = 0.0269) than that of the outer region (Table 2).

**Table 2:** Neocartilage regional mechanical properties.

Group	Region	Aggregate modulus [kPa]	Shear modulus [kPa]	Permeability [1 × 10^−15^ m^4^ N^−1^ s^−1^]	Tensile modulus [MPa]	Ultimate tensile strength [MPa]
Early OC Assembly	Outer	43.3 ± 11.7	20.5 ± 5.4^a^	13.7 ± 6.8	1.0 ± 0.2	0.4 ± 0.2
	Inner	34.1 ± 8.8	15.7 ± 4.5^b^	13.4 ± 6.3	0.8 ± 0.3	0.4 ± 0.2
Late OC Assembly	Outer	33.8 ± 5.2	24.1 ± 15.7	3.9 ± 1.1	1.1 ± 0.4^a^	0.5 ± 0.1^a^
	Inner	32.2 ± 7.4	14.9 ± 2.3	3.9 ± 0.8	0.8 ± 0.3^b^	0.4 ± 0.1^b^

Mechanical properties by region of neocartilage within each OC Assembly group. Paired *t*-tests comparing the inner vs. outer regions within the early and late OC assembly groups were performed, unless otherwise stated. Paired comparisons of aggregate modulus and shear modulus between the inner and outer regions within the late OC Assembly group were performed using a non-parametric Wilcoxon matched-pairs signed-rank.

*OC* osteochondral.

Data are presented as means ± standard deviations. Groups marked by different letters (i.e., a and b) are statistically different.

**Fig. 5:**
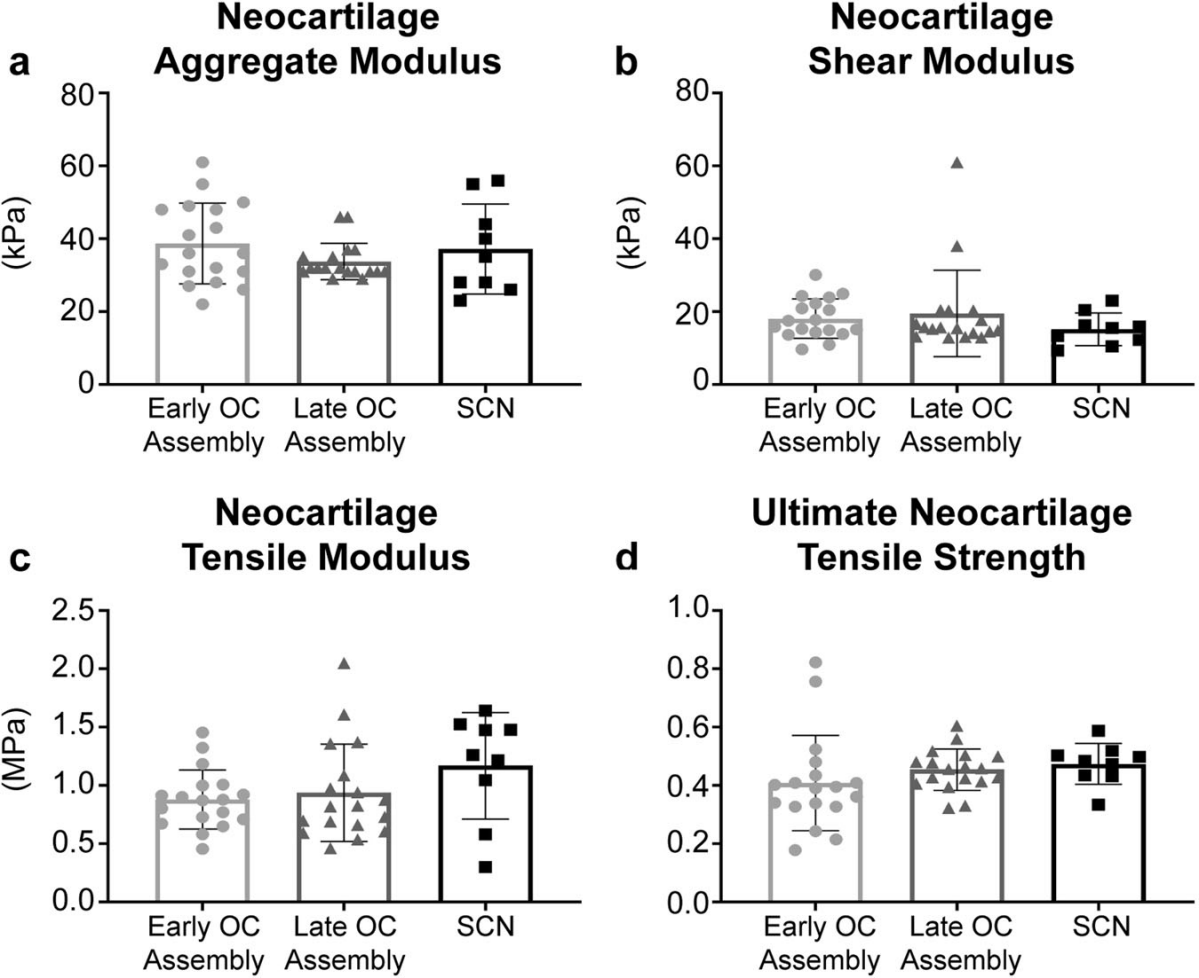
Neocartilage mechanical properties at the end of 5 weeks of culture. **a** Aggregate modulus, (**b**) shear modulus, (**c**) tensile modulus, and (**d**) ultimate tensile strength are shown. Data are presented as means with errors bars representing standard deviations. Tensile modulus data were analyzed with a one-way ANOVA with Tukey’s post hoc test. Non-parametric Kruskal–Wallis with Dunn’s post hoc tests were used to analyze aggregate modulus, shear modulus, and ultimate tensile strength. No statistical differences between groups were detected. OC osteochondral, SCN small control neocartilage.

Within the late OC assembly group, the ultimate tensile modulus and tensile strength were significantly greater (*p* = 0.0018 and *p* = 0.0325, respectively) in the outer region compared to the inner region of the neocartilage. There were no differences in aggregate modulus, shear modulus, tensile modulus, and ultimate tensile strength among the neocartilage of the Early OC Assembly group, Late OC Assembly group, and SCN (Fig. 5). Permeability of the neocartilage in the Early OC Assembly group (13.5 ± 5.4 × 10^−15^ m^4^ N^−1^ s^−1^) was significantly greater than the permeability of the neocartilage in both the Late OC Assembly group (3.9 ± 0.7 × 10^−15^ m^4^ N^−1^ s^−1^; *p* < 0.0001) and the SCN (9.2 ± 0.7 × 10^−15^ m^4^ N^−1^ s^−1^; *p* = 0.0440). In addition, the permeability of the neocartilage in the Late OC Assembly group was significantly lower (*p* = 0.0103) than that of the SCN.

**Fig. 6:**
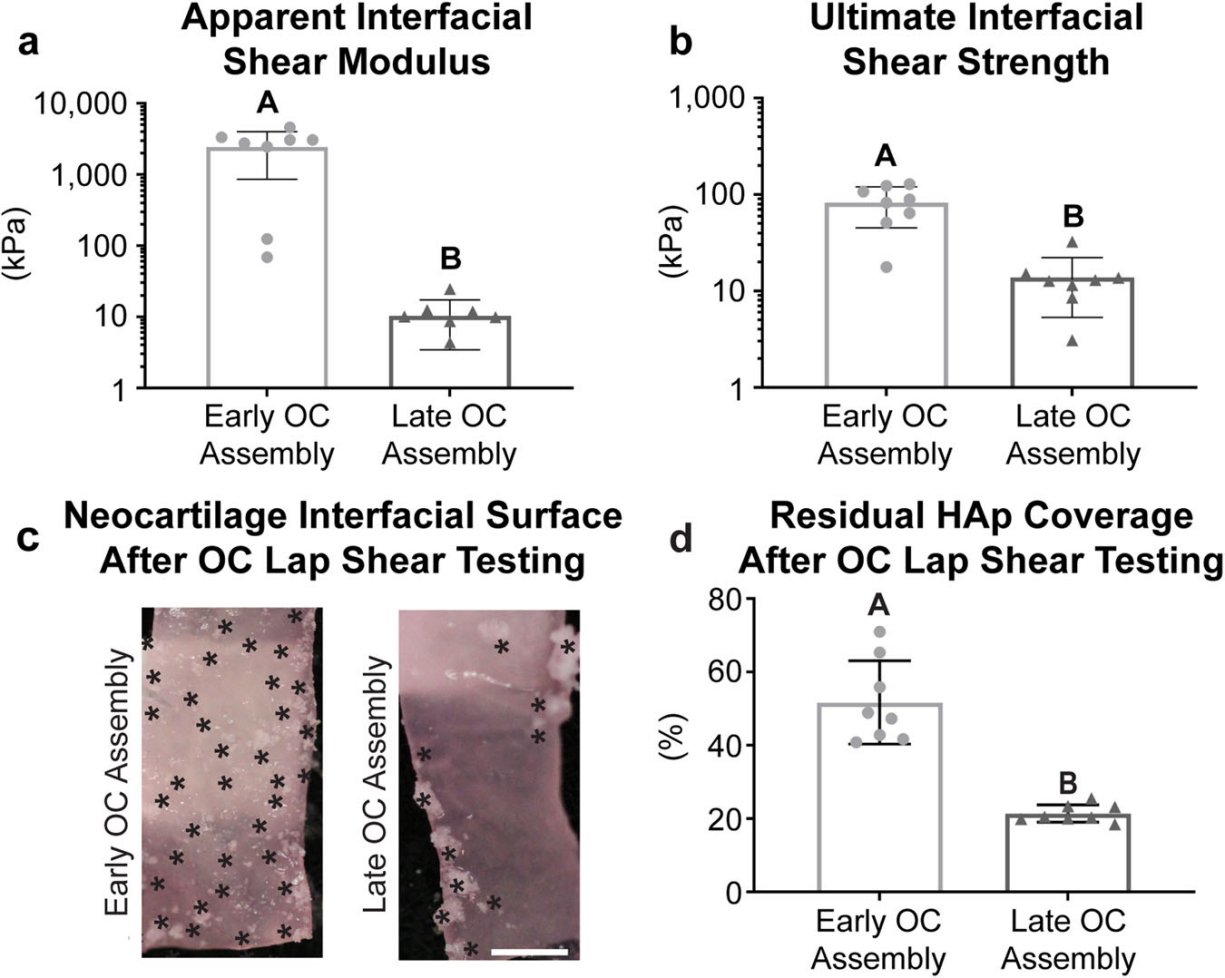
Osteochondral interfacial mechanical properties at the end of 5 weeks of culture. The (**a**) apparent interfacial shear modulus and (**b**) ultimate interfacial shear strength of the neocartilage-HAp interface, as well as (**C**) the interfacial surface of the neocartilage after lap shear testing and (**d**) the quantification of the residual HAp coverage on the neocartilage interfacial surface after lap shear testing is shown. Black asterisks mark the locations of opaque, white residual HAp particles on the pink, translucent neocartilage. Data are presented as means with errors bars representing standard deviations. The *y*-axes in (**a**) and (**b**) are on a log 10 scale. Scale bar in panel (**c**) represents 2 mm. A student’s *t*-test was used to analyze the interfacial shear modulus and residual HAp coverage, while a non-parametric Mann Whitney test was used to analyze the ultimate interfacial shear modulus. OC osteochondral, HAp hydroxyapatite.

The osteochondral constructs assembled at the early time point showed significantly superior interfacial mechanics (Fig. 6). The apparent interfacial shear modulus between the chondral and osseous phases of the osteochondral constructs was significantly greater (*p* = 0.0007) in the Early OC Assembly group (2442.4 ± 1579.1 kPa) than the Late OC Assembly group (10.4 ± 7.5 kPa). The ultimate interfacial shear strength was also significantly greater (*p* = 0.003) in the Early OC Assembly group (83.0 ± 37.7 kPa) than the Late OC Assembly group (13.9 ± 9.1 kPa).

After lap shear testing of constructs in the Early OC Assembly group, the interfacial side of the neocartilage showed roughness and contours matching the topography of the adjacent ceramic and many residually bound, fine HAp granules (Fig. 6c). The interfacial surface of the neocartilage in the Late OC Assembly group was smooth, with few large HAp granules residually adhered. The area of neocartilage covered by residual ceramic particles was quantified (Fig. 6d). The Early OC Assembly group had significantly greater coverage (51.7 ± 11.3%; *p* < 0.0001) than the Late OC Assembly group (24.8 ± 2.3%).

## Discussion

Toward engineering a unicompartmental bioprosthesis, this study successfully achieved the objectives of (1) fabricating a large, osteochondral construct consisting of self-assembled neocartilage and HAp in the size and shape of a sheep medial femoral condyle, (2) engineering a mechanically robust interface between the chondral and osseous phases, and (3) determining whether the scale-up process or exposure to HAp was detrimental to neocartilage mechanical properties. The main hypotheses of this study were that assembling the chondral and osseous phases prior to neocartilage maturation would result in enhanced interfacial mechanical properties and that the neocartilage of the chondral phase would have homogeneous functional properties on par with SCN constructs. Both hypotheses were shown to be correct. Assembling the chondral and osseous phases before neocartilage maturation, at day 4 (Early OC Assembly), versus at day 10 (Late OC Assembly), increased neocartilage interdigitation depth into the HAp pores by 244%, increased interdigitation frequency by 438%, and, importantly, increased the apparent shear modulus and the ultimate shear strength of the osteochondral interface by 243-fold and 4.9-fold, respectively. The superior interfacial properties achieved with the Early OC Assembly group were obtained without sacrificing neocartilage functional properties. This was demonstrated by the lack of differences in functional properties between regions of the neocartilage within each assembly group, between neocartilage from both OC Assembly groups, and between the neocartilage of both OC assembly groups and 5 mm-diameter neocartilage control constructs. Importantly, the osteochondral constructs in the Early OC Assembly group maintained shape fidelity over the total culture duration of 5 weeks. This study showed the fabrication of large, anatomically shaped, osteochondral constructs with robust interfacial mechanical properties native-like neocartilage interdigitation.

This study demonstrates that early assembly (at day 4) of the chondral and osseous phases increased neocartilage interdigitation depth and frequency into the HAp and increased interfacial mechanical properties. The maturational state of the neocartilage at OC assembly is likely responsible for the enhanced interdigitation. During the self-assembling process, there are distinct phases: (1) seeding of a high density of cells in a non-adherent environment, (2) integrin and N-cadherin mediated cell-to-cell interactions and tissue formation^33^, (3) production of immature cartilage ECM, and (4) ECM maturation^35^. In this study, time points of osteochondral assembly, which correspond to different phases of the self-assembling process, were selected to determine if neocartilage maturation played a significant role in osteochondral interfacial mechanics. The Early OC Assembly time (day 4) corresponds to neocartilage in phase 3. The Late OC Assembly time (day 10) corresponds to neocartilage in phase 4. In self-assembled neocartilage derived from primary bovine ACs, the processed form of collagen II appeared after 8 h^33^, and total collagen content per construct, as well as construct thickness, increased throughout the 8-week culture period^34^. However, collagen/WW decreased after 7 days of culture^34^ because of a concomitant increase in GAG content and construct hydration after day 4^34^. Therefore, regarding key maturational differences between day 4 and 10, it is likely that when osteochondral assembly occurred on day 4, more collagen within the matrix was spatially available to adsorb to the HAp. Enhanced ECM adsorption to HAp is known to increase HAp adhesion strength with soft tissue^36^. Additionally, OC assembly at day 4 allowed for more deposition of the cartilaginous matrix within the ceramic pores over the course of ECM synthesis, compared to the amount of matrix deposition that accumulated after assembly at day 10. This resulted in a 244% increase in depth of neocartilage interdigitation and a 438% increase in the frequency of interdigitation (Fig. 3c, d). It also enhanced neocartilage adherence to the ceramic, as demonstrated by 2.4 times more residual HAp coverage on the neocartilage in the Early OC Assembly group compared to the Late OC Assembly group (Fig. 6c, d). The difference in adherence of the chondral phase to the osseous phase between OC Assembly groups is grossly evident by the presence of deformations or lifted areas in the Late OC Assembly group compared to the homogeneous and complete adherence observed in the Early OC Assembly group (Fig. 2). The increased chondral interdigitation depth and frequency, as well as and adherence to the osseous phase likely were the primary factors that caused the 243-fold greater apparent interfacial shear modulus and 4.9-fold greater shear strength (Fig. 6). These data show that enhancing neocartilage matrix deposition and adherence within the pores of the osseous phase in an OC construct is important to achieve robust interfacial properties.

The difference in the amounts of HAp that remained adherent on the neocartilage after lap shear testing of the assembly groups serves to inform the mode of failure at the osteochondral interface. Failure within either the neocartilage or HAp phases represents a cohesive mode of failure; failure at the interface between the phases represents an adhesive mode of failure. For both the Early and Late OC Assembly groups, the neocartilage ultimate tensile strength exceeded the ultimate interfacial shear strength, indicating that cohesion within the neocartilage phase did not play a role in the interfacial mechanical properties. The mode of failure of the Late OC Assembly group may be classified as mostly adhesive because the neocartilage was cleanly separated from the ceramic, with few residual HAp particles remaining on the neocartilage (Fig. 6c). The mode of failure at the osteochondral interface in the Early OC Assembly group may be classified as mixed (adhesive and cohesive) because residual HAp particles remained on the underside of the undamaged neocartilage phase after lap shear testing (Fig. 6c). This indicates the adhesive or interfacial strength exceeded the cohesive strength of the osseous phase. The ultimate interfacial shear strength of the osteochondral interface in the Early OC Assembly group was 82 kPa (Fig. 6), over 12-fold greater than what has been achieved with 5 mm-diameter osteochondral constructs composed of HAp and bone marrow mesenchymal stem cells within polyethylene glycol diacrylate^37^ and over threefold greater than with 6 mm-diameter osteochondral constructs composed of native trabecular bone with a tissue-engineered zone of calcified cartilage^38^. In addition, the shear strength was on par with what has been achieved in 10 × 10 mm osteochondral constructs composed of polyethylene glycol and β-tricalcium phosphate of comparable porosity to the HAp in this study^39^ and on par with what we have previously achieved in 5 mm-diameter, small osteochondral constructs containing self-assembled neocartilage and HAp^32^. Because the depth of interdigitation in the Early OC Assembly group was on par with that of native tissue, future studies to improve interfacial properties should focus on factors other than interdigitation depth.

Manipulating ceramic pore characteristics or enhancing the transitional gradient between chondral and osseous phases may further improve osteochondral interfacial mechanical properties. For example, interfacial strength may be improved by manipulating the HAp pore size or pore tortuosity to increase the mechanical interlocking of the phases^39^. Interfacial strength may also arise from collagen fibers bridging the two phases, analogous to Sharpey’s fibers (i.e., collagen fibers that extend from soft tissue into bone). These collagen fibers have been observed in articular cartilage and their formation may require the development of a calcified cartilage layer^40^. A sudden transition between materials of greatly different mechanical properties can create stress concentrations. Thus, engineering a gradient of tissue properties, such as inducing development of a calcified cartilage layer, can also potentially enhance mechanical properties of the osteochondral interface^41–43^. While many studies have focused on enhancing the adhesion and integration of the chondral and osseous phases, the mixed mode of lap shear failure in the Early OC Assembly group also suggests that it is also important to enhance the cohesive strength of the osseous phase alone, potentially by incorporating high tensile-strength materials, such as collagen.

The goal of osteochondral tissue engineering is to create a cartilage-bone unit with mechanical, biochemical, and morphological properties, particularly the transitional calcified cartilage zone, matching those of native osteochondral tissue. A functionality index (FI) has been previously used to compare tissue-engineered neocartilage mechanical and biochemical properties to those of healthy native tissue. The FI provides a summary of how closely the engineered tissue achieves native tissue properties; an FI of 1 represents 100% of native tissue properties. It would be advantageous to modify this FI to evaluate the biomimicry of osteochondral tissue by adding an interfacial shear modulus parameter, for example. However, complete biomimicry may not be necessary to elicit a healing response. It has been shown that, compared to empty defects, neocartilage achieving an FI = 0.42 elicited excellent healing characterized by significantly more defect closure and stiffer repair tissue when implanted into a partial-thickness defect in the temporomandibular joint discs of Yucatan minipigs in vivo. Furthermore, the implant prevented the progression of osteoarthritic degenerative changes seen in the empty defects. Therefore, osteochondral constructs may have the potential to also elicit a healing response without achieving an FI = 1. While the goal of osteochondral tissue engineering is to generate a completely biomimetic cartilage-bone unit, complete biomimicry may not be necessary for healing.

Limitations in nutrient diffusion result in neocartilage with poor functional properties and have been a major obstacle to engineering large neocartilage constructs. Importantly, this study overcame this hurdle. Within the Early OC Assembly group, the neocartilage phase did not show regional differences in biochemical content or mechanical properties (Tables 1 and 2). In the Late OC Assembly group, the outer region of the neocartilage phase had significantly more GAG/DNA, collagen/WW, collagen/DW, and collagen/DNA. However, there were no regional differences in the neocartilage mechanical properties. Gross examination and histology of the chondral phase of OC construct from both assembly groups indicated that the tissue was homogeneous, and limitations in nutrient diffusion were not evident (Figs. 2 and 3a). This is further supported by the lack of differences in neocartilage mechanical properties between the OC assembly groups (Table 2), as well as between either OC assembly group and the SCN group (Fig. 5). The lack of regional differences in neocartilage functional properties suggests that nutrient diffusion was not impeded by construct scale-up or osteochondral culture. The functional properties of the chondral phase were also on par with those previously achieved by joAC-generated self-assembled neocartilage in small osteochondral constructs containing HAp^32^. It has been shown that large scaffold-based engineered neocartilage constructs have inferior properties compared to smaller constructs^22,23^, likely due to diffusion limitations. However, these data further support previous findings that the neocartilage self-assembling process may be scaled up to achieve large constructs without deterioration of functional properties^24^. These results show that osteochondral constructs containing self-assembled neocartilage may also be scaled-up, while preserving the functional properties of the chondral phase.

Another major obstacle to successfully generating osteochondral constructs in clinically relevant sizes and shapes is the maintenance of shape fidelity over the duration of in vitro culture. Shape fidelity of engineered implants is critical to ensure normal joint loading and to prevent stress concentrations that can cause neocartilage delamination or destruction in situ. While custom shapes may be achieved by casting hydrogels containing embedded cells^21–23^ or cell-seeding 3D-printed scaffolds^44^ to guide neocartilage formation, the limitations of exogenous materials in cartilage engineering are well-known^35^. Maintaining shape fidelity in scaffold-free neocartilage is more difficult because cell-mediated tissue contraction, cell migration, and matrix production may alter construct shape. These effects are more profound with large scaffold-free neocartilage constructs compared to small constructs because of their increased cellularity and cellular traction, which result in more internal residual stress and tissue warping. For example, scale-up of 5 mm-diameter self-assembled neocartilage constructs to 25 mm-diameter constructs resulted in wrinkles and deformities not previously observed in the small constructs^24^. However, neocartilage treatment with cyto D, an inhibitor of actin polymerization, reduced warping by inhibiting stress fiber contraction within cells and residual stress within the engineered neocartilage^24^. Importantly, within this study, shape fidelity of the chondral phase in the Early OC Assembly group was maintained over the 5-week culture period, as demonstrated by its smooth, homogeneous appearance (Fig. 2) and its consistent, deep, and frequent interdigitation into the osseous phase (Fig. 3b–d). However, points of neocartilage delamination and deformation were seen in the Late OC Assembly group. Because both groups underwent the same treatment regimen, which included cyto D, this implies that other factors, such as neocartilage maturational state at shape formation, also affect the maintenance of shape fidelity.

The use of a juvenile allogeneic approach, as envisioned in this study, offers translational advantages over an autologous approach. Autologous approaches for cartilage repair are limited by the scarcity of healthy donor cartilage and donor site morbidity in an already damaged joint. Patients must undergo two surgeries, one to harvest donor cartilage and a second to receive the cartilage implant. Furthermore, autologous treatments use chondrocytes from mature (>18 years old) donors^45^. Therefore, these treatments are limited by the quality of a patient’s own donor cartilage, which is known to be highly variable and to deteriorate with age and injury. In contrast, juvenile (<13 years old) ACs possess superior expansion and cartilaginous matrix formation capacities compared to adult ACs or stem cells^46–48^. An allogeneic approach alleviates donor tissue scarcity and eliminates donor site morbidity and biological variability. Allogeneic donor cartilage must undergo extensive donor screening and qualification per FDA guidance^49^, which results in a well-characterized cell source and a consistently high-quality product with minimal concern for disease transfer. Furthermore, allogeneic cartilage repair products do not have to be patient-matched, unlike autologous therapies, which eliminates the potential of a patient receiving an implant meant for another person. These benefits enable the future production of functional, off-the-shelf cartilage repair implants in clinically relevant sizes and eliminate the need for multiple patient surgeries.

This study sought to engineer osteochondral constructs in a clinically relevant size and shape with robust interfacial mechanical properties. Specifically, constructs were generated to replicate the medial femoral condyle of adult sheep, toward the eventual fabrication of a unicompartmental bioprosthesis. The chondral phase consisted of scaffold-free, self-assembled neocartilage and the osseous phase consisted of porous HAp ceramic capable of integration with native bone. Importantly, scale-up and exposure to HAp did not adversely affect the mechanical properties of the neocartilage. In addition, diffusion limitations that have limited the successful fabrication of large scaffold-based neocartilage constructs were not observed. Early assembly of the chondral and osseous phases to form osteochondral constructs significantly increased the interfacial mechanical properties; it also increased neocartilage interdigitation depth within the osseous phase to a degree on par with native osteochondral tissue. Construct shape fidelity was maintained over the entire duration of the 5-week culture, overcoming another common limitation to engineering functional neocartilage of clinically relevant sizes and shapes. Importantly, this work shows that the self-assembling process can be used to fabricate clinically sized, anatomically shaped osteochondral constructs. By using the translationally relevant allogeneic juvenile sheep model, these outcomes help elucidate a strategy toward engineering a bioprosthesis for the repair of articular lesions encompassing an entire condylar surface in a manner similar to existing, synthetic unicompartmental prostheses.

## Methods

### Overview

Following a series of pilot studies examining the seeding of additional cells between the chondral and osseous phases, adsorption of exogenous collagen to the ceramics prior to osteochondral assembly, and the application of high concentration bioactive agents, such as LOXL2, it became apparent that the most significant factor affecting the osteochondral interface was the timing of OC assembly. Thus, this study examined the effects of early and late osteochondral assembly at day 4 and 10, respectively, of neocartilage maturation.

### Knee 3D modeling and mold fabrication

**Fig. 7:**
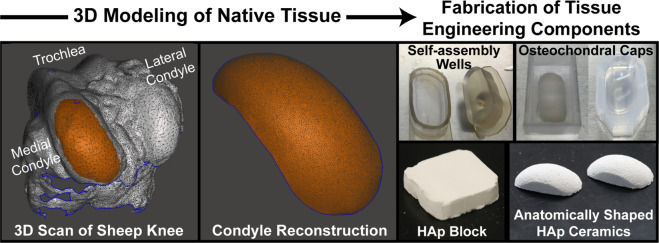
Fabrication of HAp blocks and self-assembling wells. A laser-scanned, 3D model of ovine knees was used to create an anatomical surface of the medial femoral condyle. With this surface, appropriately shaped agarose molds and HAp ceramics were made. Two agarose molds were used for culturing the neocartilage: (1) self-assembly wells were made by adding agarose to a flat-bottom, 3D-printed well and placing a negative cast on top; (2) osteochondral caps were made by adding agarose to a rectangular well containing a plastic reconstruction of the sheep medial condyle. HAp ceramics in the shape and size of the medial femoral condyle were made by machining a large HAp monolith to appropriate shapes with a CNC mill. HAp hydroxyapatite.

Stifle joints from skeletally mature, Rambouillet Suffolk cross sheep were obtained from a local abattoir (Superior Farms, Dixon, CA). The muscle and fat were removed from the distal femur (femoral condyles and trochlea), and the joints (*n* = 3) were laser-scanned (NextEngine) to obtain 3D models of the knees. The resulting models were imported into MeshMixer (Autodesk Inc.) for processing. The models were combined to generate a single representative model, and the surface of the medial condyle was manually selected and isolated using a trimming tool. Scanning defects on the surface were repaired, and the boundary of the isolated region was smoothed using available software functions (Fig. 7).

To make anatomically shaped, flat-bottom agarose wells for self-assembly, a two-part plastic mold system consisting of a well and a negative cast (self-assembly wells; Fig. 7) was designed in Autodesk Inventor and 3D-printed (Objet System, Stratasys; MED610 material). The mold system was designed such that the outline of the agarose well-matched a two-dimensional projection of the outline of the modeled medial femoral condyle and had inner dimensions of 31 × 14 mm.

Another plastic mold system was designed to make shape-specific agarose caps to confine the engineered neocartilage on top of the HAp ceramic during osteochondral assembly (osteochondral caps; Fig. 7). In MeshMixer, the surface of the medial condyle was extruded to create a condyle-shaped dome, which was then 3D-printed (MED610 material). This dome was designed to be 2 mm shorter than the height of the HAp ceramics, creating a 2 mm offset between the bottom of the petri dish and the agarose caps to ensure the caps would seat properly on the osteochondral constructs during culture.

### Fabrication of HAp ceramics

HAp ultrafine microparticles (22.5 g, 2 µm-diameter; Himed) were combined with 10% (wt/v) Brij (Sigma) (27 mL) in ultrapure water and polyethylenimine (Sigma) (4 mL) and vortexed overnight to form a foam. The foam was thoroughly mixed with glycerol diglycidyl ether (Sigma) (1 mL) and scooped into rectangular silicon molds, sealed in plastic bags, and placed in a 40 °C oven overnight to allow the blocks to solidify. Promptly after 18 h, the HAp blocks were gently removed from the molds, again sealed in plastic bags, and frozen for at least 2 h at −80 °C. After the blocks were fully frozen, they were lyophilized for 2 days to stabilize them. The HAp blocks were then milled into a dome-shaped geometry to mirror the ovine medial femoral condyle. To do so, tool paths were developed in the computer aided machining (CAM) software (Esprit), and the blocks were milled using a Computer Numerical Control (CNC) ball mill (1/8 in.; Fig. 7). The resulting anatomically shaped HAp ceramics were sintered in an oven (Nabertherm P330) using the following scheme: The temperature increased from room temperature (25 °C) to 600 °C at 1 °C/min. This temperature was held for 1 h to burn off the epoxy within the ceramics. The temperature was then increased to 1310 °C at 4 °C/min and held for 8 h before returning to room temperature.

### Chondrocyte isolation

joACs were isolated from the femoral condyles and trochlear groove of Rambouillet Suffolk cross sheep (*n* = 6) obtained from an abattoir (Nature’s Bounty Farms, Dixon, CA) within the same day of animal sacrifice. Cartilage was minced into 1–2 mm^3^ cubes and washed two times with chondrogenic medium (Dulbecco’s Modified Eagle Medium; DMEM, containing 4.5 g/L glucose with GlutaMAX; Gibco, 1% (v/v) PSF; Lonza, 1% (v/v) ITS; BD Biosciences, 1% (v/v) NEAA; Thermo Fisher Scientific, 100 μg/mL sodium pyruvate; Thermo Fischer Scientific, 50 μg/mL ascorbate-2-phosphate; Sigma, 40 μg/mL L-proline; Sigma, and 100 nM dexamethasone; Sigma). Minced cartilage was digested with 500 units/mL collagenase type 2 (Worthington Biochemical) in chondrogenic medium + 3% (v/v) fetal bovine serum (FBS; Atlanta Biologicals) for 18 h at 37 °C and 10% CO_2_ on an orbital shaker. The digested cartilage solution was then strained through a 70 μm strainer to isolate the chondrocytes. The chondrocytes were counted and cryopreserved in DMEM + 20% (v/v) FBS + 10% (v/v) DMSO (Sigma).

### Chondrocyte expansion and redifferentiation

Primary (P0) joACs were thawed and seeded into T-225 flasks at 25,000 cells/cm^2^. Cells underwent chondrotuning expansion in the monolayer to passage 4 (P4) in chondrogenic medium with TFP supplementation (1 ng/mL TGF-β1, 5 ng/mL FGF-2, and 10 ng/mL PDGF-BB; all from Peprotech) at 37 °C and 10% CO_2_. For the first 24 h of each passage, 10% (v/v) FBS was supplemented to promote cell adhesion. After 24 h, the FBS concentration was lowered to 3% (v/v) and maintained for the rest of the passage duration. Medium was changed every 2–3 days. Cell monolayers were cultured for an additional 4 days after 95% confluence was reached. Cell sheets were lifted by incubation with 0.25% trypsin/EDTA (Invitrogen) for 20 min. The sheets were then digested with 500 units/mL collagenase type 2 in DMEM + 3% (v/v) FBS for 45–60 min. Cells were filtered through a 70 µm cell strainer, washed three times with DMEM, counted with a hemocytometer, and either passaged again or placed into aggregate culture.

P4 joACs underwent aggregate redifferentiation culture to reinstate their chondrogenic phenotype post-expansion^50,51^. Briefly, 10 cm-diameter petri dishes were coated with molten 2% (wt/v) agarose to prevent cell adhesion. In each dish, 25 million P4 redifferentiated (P4R) joACs were placed in chondrogenic medium (25 mL) supplemented with TGB (10 ng/mL TGF-β1, 100 ng/mL GDF-5, and 100 ng/mL BMP-2; all from Peprotech). Dishes containing cells were placed on an orbital shaker at 55 rpm for 24 h and then cultured statically for an additional 13 days. The medium was exchanged every 2–3 days. On day 14, cell aggregates were digested in 0.25% trypsin/EDTA for 20 min, followed by 500 units/mL collagenase type 2 in DMEM + 3% FBS for 1–2 h. Cells were filtered through a 70 µm cell strainer, washed three times in DMEM, counted, and self-assembled into neocartilage constructs.

### Neocartilage construct self-assembly and culture prior to osteochondral assembly

Anatomically shaped self-assembly wells (3.78 cm^2^) were made by adding of molten 2% (wt/v) agarose (4 mL) into the two-part well makers described in the Knee 3D Modeling and Mold Fabrication Section. After solidification of the agarose, the wells were removed and placed in chondrogenic medium. The medium was exchanged daily for 5 days to ensure saturation of the agarose. The agarose wells were housed in 10 cm-diameter, 2.5 cm-tall petri dishes. Large, scaffold-free neocartilage constructs, formed via the self-assembling process^52^, were generated by seeding 38.6 million P4R joACs (10.2 million cells/cm^2^) in chondrogenic medium (2 mL) containing 2 µm cyto D (Enzo Life Sciences) and 10 ng/mL TGF-β1 into the anatomically shaped agarose wells (seeding is defined as *t* = 0 days). After 4 h, chondrogenic medium (40 mL) containing 2 µm cyto D and 10 ng/mL TGF-β1 was carefully added to each petri dish containing a newly formed construct. Neocartilage received cyto D treatment from days 0 to 3 of culture and 10 ng/mL TGF-β1 was maintained through the duration of the culture. The medium was exchanged daily until osteochondral construct assembly.

SCN constructs, 5 mm in diameter (0.196 cm^2^), were also seeded^53^. Briefly, agarose wells were made by inserting 5 mm-diameter stainless steel posts into molten 2% (wt/v) agarose (1 mL) in a 48-well plate. After solidification of the agarose, the posts were removed, and the wells were filled with chondrogenic medium. Medium was exchanged daily for 5 days to ensure saturation of the agarose. In each well, 2 million P4R joACs (10.2 million cells/cm^2^) were seeded in a 50 µL chondrogenic medium containing 2 µm cyto D and 10 ng/mL TGF-β1. This seeding density (number of cells per cm^2^) was directly scaled by surface area from the seeding density of the large neocartilage constructs. An additional 450 µL of chondrogenic medium containing 2 µm cyto D and 10 ng/mL TGF-β1 was added to each well 4 h after seeding. As with the large constructs, Cyto D was maintained in the medium from days 0 to 3 and 10 ng/mL TGF-β1 was maintained through the duration of the culture. The medium was exchanged daily until unconfinement at day 2, and exchanged every other day following that, for the duration of the 35-day culture period. SCN constructs were not exposed to HAp.

### Osteochondral construct assembly and culture

**Fig. 8:**
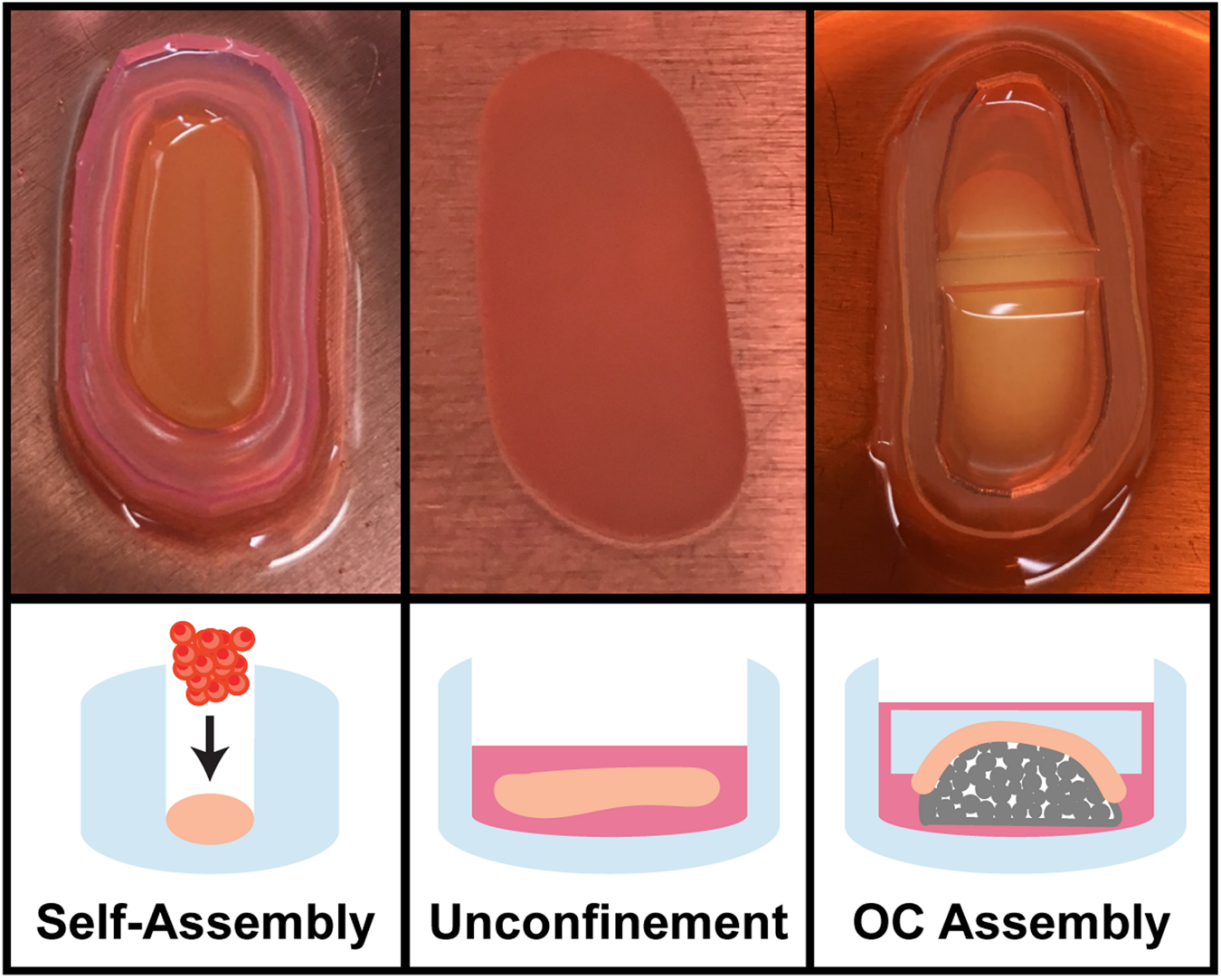
Osteochondral assembly. Passaged joACs were seeded into flat-bottom agarose wells (31 × 14 mm) for self-assembly (*t* = day 0). At day 2, the neocartilage was unconfined from the wells and cultured as a free-floating construct. After OC assembly of the neocartilage and HAp occurred at day 4 or day 10, an agarose cap was placed on top of the osteochondral construct. OC osteochondral.

Osteochondral caps (described in the Knee 3D Modeling and Mold Fabrication) were formed by placing the 3D-printed condyle-shaped domes in the center of 3D-printed rectangular-shaped boxes (Fig. 7) and filling the remaining volume with molten 2% (wt/v) agarose. After solidification of the agarose, the agarose caps were separated from the domes and boxes and incubated in chondrogenic medium for 5 days prior to use to ensure saturation. At either day 4 (Early OC Assembly group) or day 10 (Late OC Assembly group) after neocartilage self-assembly, the osteochondral constructs were assembled (Fig. 8). Immediately prior to OC assembly, 10 HAp ceramics were submerged in of 10% FBS in DMEM (10 mL) under vacuum for 10 min, and then rinsed once in DMEM. To assemble the osseous (HAp ceramics) and chondral (self-assembled neocartilage) phases, the shape-specific agarose caps were inverted so the dome-shaped reservoir faced up, and the neocartilage constructs were draped over the reservoir. The ceramics were inverted (dome side down), placed on the neocartilage within the agarose reservoir, and seated into the contours of the cap. The entire osteochondral unit (ceramic, neocartilage, and agarose cap) was then inverted to the upright position (with the agarose cap on top of the neocartilage and ceramic). A 3D-printed (Objet System, Stratasys; MED610 material), 1 g mass was applied to the top of each agarose cap/osteochondral unit to ensure full contact between the osseous and chondral phases. At day 15, a 20 g stainless steel mass (316 stainless steel; McMaster-Carr) was applied to further encourage neocartilage interdigitation into the HAp ceramic. On day 21, the 20 g mass was removed and replaced with a 1 g mass to allow for neocartilage growth. Osteochondral constructs were maintained with the 1 g mass for the rest of the duration of the 35-day culture period. Post-osteochondral assembly, medium was exchanged every 5–6 days throughout the culture period.

### Bioactive stimulation regimen

**Fig. 9:**
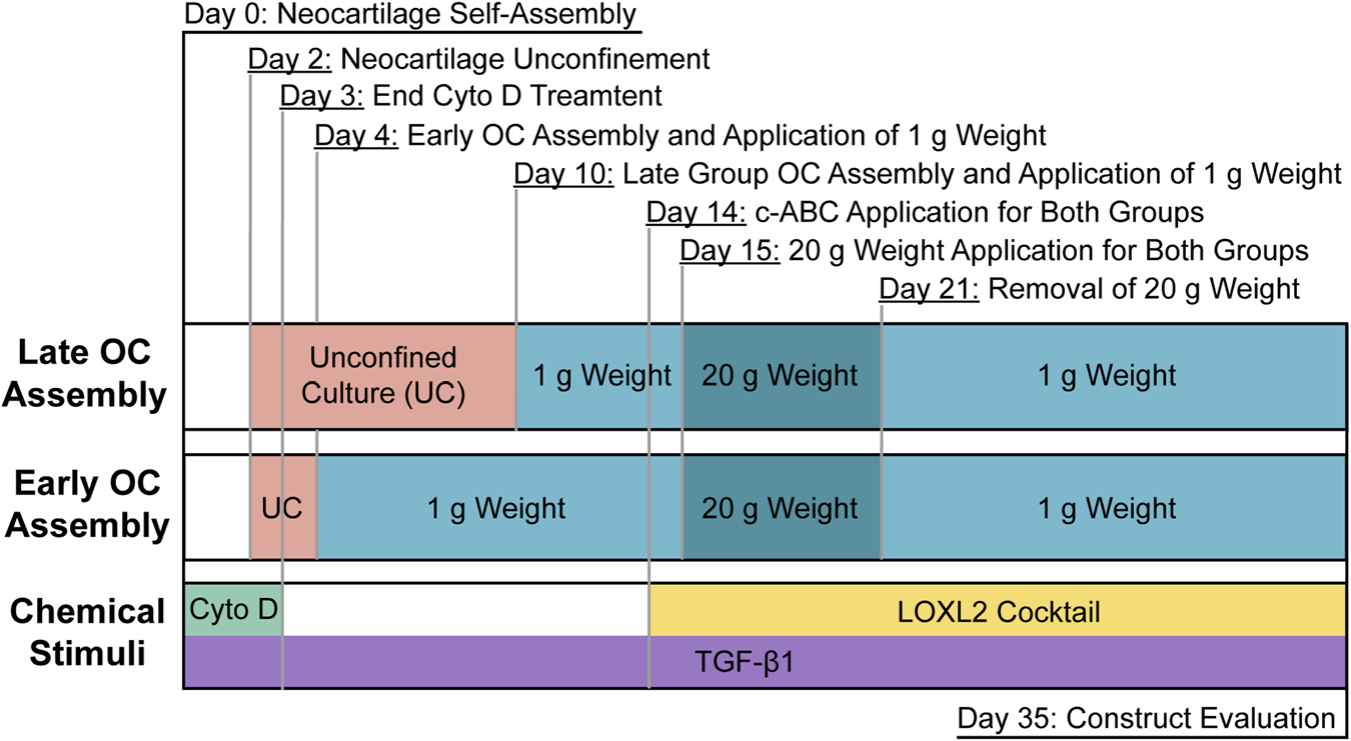
Culture and treatment timeline. OC osteochondral, Cyto D cytochalasin D, c-ABC chondroitinase-ABC, LOXL2 lysyl oxidase-like 2 protein.

Osteochondral constructs and SCN constructs were cultured in chondrogenic medium under a defined chemical stimulation regimen (Fig. 9). As described above, 2 µM cyto D was added to the medium at neocartilage self-assembly (day 0) until day 3 and TGF-β1 was added at 10 ng/mL throughout the entire duration of culture (days 0–35). c-ABC (Sigma) at 2 U/mL was applied for 4 h on day 14. After c-ABC application, constructs were washed with chondrogenic medium containing 1 mM zinc sulfate (Sigma) three times to deactivate and remove residual c-ABC. A LOXL2 cocktail was applied immediately following c-ABC treatment and maintained continuously days 14–35. The cocktail was comprised of 0.15 µg/mL LOXL2 (Genway Bio), 1.6 µg/mL copper sulfate (Sigma), and 146 µg/mL hydroxylysine (Sigma).

### Gross morphological analysis

Osteochondral constructs were examined grossly, and pictures were taken prior to portioning of the construct for histological, biochemical, and mechanical evaluations. Images were taken from distinct samples.

### Histological evaluation and interdigitation quantification

Neocartilage was gently removed from the HAp ceramic with a spatula or a razor when necessary and fixed in 10% neutral buffered formalin (NBF). Fixed neocartilage was embedded in paraffin and cut to 5 µm-thick sections. Sections were stained with hematoxylin and eosin (H&E) to show tissue morphology, Safranin O and Fast Green to show sulfated GAG deposition, and picrosirius red to show collagen deposition, as well as von Kossa and alizarin red to show calcification and mineralization. Native juvenile ovine articular cartilage sections were used as controls for H&E, Safranin O/Fast Green, and picrosirius red stains. Native juvenile ovine costal (rib) cartilage sections were used as controls for alizarin red and von Kossa stains. Immunohistochemistry (IHC) was also performed to visualize collagen I (ab34710, 1:500 dilution, Abcam) and collagen II (ab34712, 1:4000 dilution, Abcam). Decalcified native juvenile ovine osteochondral tissue sections were used for IHC controls. Full thickness osteochondral constructs from both groups, as well as native juvenile ovine osteochondral tissue samples, were also fixed in 10% NBF, serially dehydrated in ethanol, and embedded in Technovit 7200 resin (Exakt). Sections were cut from the block, polished to 40 µm thick, and stained with toluidine blue to show the osteochondral interface.

The junction of engineered neocartilage and HAp, as well as the junction of native cartilage and native subchondral bone were visualized and defined via microscopic examination of the toluidine blue histology images. To measure the depth of neocartilage interdigitation within the osteochondral constructs and the depth of native cartilage interdigitation within native osteochondral tissue, the maximum depths of cartilage extending beneath the neocartilage-HAp or native cartilage-bone junctions were measured in ImageJ. To measure the frequency of interdigitation in the osteochondral constructs and native osteochondral tissue, 1 mm long regions of interest and the top border of ceramic or subchondral bone were defined on the toluidine blue-stained histology images of the osteochondral interface. The number of times the neocartilage or native cartilage proceeded below the defined ceramic/bone border was counted. All histological evaluations were performed on distinct samples.

### Biochemical evaluation

Neocartilage samples were removed from the outer and inner regions of the ceramic for histological examination from both OC Assembly groups. Samples from the outer region were taken from the outer circumferential periphery of the neocartilage, no more than 3 mm away from the edge (Fig. 1). Samples from the inner region were taken from the exact middle of the neocartilage. All samples were weighed to obtain wet weights WW, lyophilized for 3 days, and weighed again to obtain dry weights. Samples were then digested with 125 µg/mL papain (Sigma), 5 mM N-acetyl-L-cysteine (Sigma), and 5 mM EDTA (Acros Organics) in phosphate buffer overnight at 60 °C. Sulfated GAG content was measured using the Blyscan GAG Assay kit (Biocolor). DNA content was measured with a Picogreen assay. Total collagen content was determined by measuring hydroxyproline content using a modified chloramine-T colorimetric assay^54^. A standard curve was generated using a Sircol collagen standard (Biocolor). GAG and collagen content were normalized to wet weight, dry weight, and DNA content. All biochemical measurements were performed on distinct samples.

### Mechanical evaluation and quantification of residual HAp coverage

Creep indentation compressive testing was performed on full-thickness osteochondral constructs from in the outer and inner regions of both OC Assembly groups and on 5 mm-diameter control neocartilage constructs. Samples were collected from the outer and inner regions for biochemical analysis. A 0.8 mm-diameter porous, stainless steel tip under a 0.7 g mass was applied to the neocartilage surface of samples to achieve 10–15% neocartilage strain. As previously described, a semi-analytical, semi-numerical, linear biphasic model followed by a finite element model was used to obtain the neocartilage aggregate and shear modulus values from the experimental data^55^.

For uniaxial tensile testing, neocartilage was gently pried up from the HAp ceramic using a blunt spatula, and dog bone-shaped samples were cut from both the inner and outer regions. Dog bone-shaped samples were also cut from the 5 mm-diameter control neocartilage constructs. Samples were glued to paper tabs with gauge lengths of 1.8 mm. The paper tabs were gripped outside the gauge length into a mechanical testing system (TestResources) and pulled at 1% of the gauge length/second until sample failure. The cross-sectional area of each sample was calculated in ImageJ from front- and side-view photographs of the dog bone and used to generate stress–strain curves. The neocartilage tensile modulus was obtained from the linear region of the stress–strain curve and the neocartilage ultimate tensile strength was defined as the maximum stress reached.

The mechanical properties of the neocartilage-ceramic interface of the osteochondral constructs were evaluated with lap shear testing. The ceramic side of full-thickness osteochondral samples was glued to a flat, wooden popsicle stick to provide an immobile anchor. A paper tab was glued to the neocartilage side of the osteochondral samples. The wooden stick and paper tab were loaded into offset grips (to eliminate the creation of a bending moment) in the mechanical testing system (TestResources) such that the neocartilage-ceramic interface was parallel to the direction of strain. The sample was pulled apart at a rate of 1 mm/min until failure. Sample surface areas were calculated from images of the samples and used to generate stress–strain curves. The apparent interfacial shear modulus of the osteochondral samples was obtained from the linear region of the stress–strain curve, and the ultimate interfacial shear strength of the osteochondral samples was obtained from the maximum stress reached.

After lap shear testing, the area covered by residual HAp on the interfacial side of the neocartilage was measured. In ImageJ, 6 × 6 mm regions of each neocartilage surface were randomly selected, and the surface area of that region was measured. Because the ceramic has a porosity of 55%, the contact area of the neocartilage was calculated as 45% of the total area. The cumulative surface area of the remaining HAp particles within that region was also measured. Residual HAp coverage was calculated by normalizing the cumulative surface area of residual HAp particles by the corrected surface area of the interfacial contact area of the region. All mechanical measurements were performed on distinct samples.

### Statistical analysis

Sample size was determined to be *n* = 8 per group based on a power analysis using pilot study data (means and standard deviation) with thresholds of power = 0.8 and alpha = 0.05 using JMP Pro 14 (SAS). All other analyses were performed in Prism (GraphPad Software). Outliers were determined by using a ROUT outlier test and were removed before further statistical analysis. A Kolmogorov–Smirnov test was used to test all data for normal distribution. For two-sample paired analyses, such as the analysis of regional biochemical and mechanical data (inner vs. outer regions) of each construct within the Early and Late OC Assembly groups, a paired *t*-test (two-tailed/sided; *p* < 0.05) or a Wilcoxon matched-pairs signed-rank test was performed for normally distributed or non-normally distributed data, respectively. Regional biochemical and mechanical data were then pooled to yield representative values for each construct. For two-sample, unpaired analyses, such as the comparison of the pooled biochemical data between OC Assembly groups, a Student’s *t*-test (unpaired, two-tailed/sided, *p* < 0.05) or a Mann Whitney test (*p* < 0.05) was used for normally distributed and non-normally distributed data, respectively. For analyses among more than two groups, a one-way analysis of variance (ANOVA; *p* < 0.05) with Tukey’s post hoc multiple comparisons test or a Kruskal–Wallis (*p* < 0.05) test with Dunn’s multiple comparison post hoc test were performed for normally distributed and non-normally distributed data, respectively. Data are presented as means with errors bars representing standard deviations. Groups marked by different letters are statistically different. The statistical tests performed for each data set displayed in the figures are noted in the captions.

### Reporting summary

Further information on research design is available in the Nature Research Reporting Summary linked to this article.

## Supplementary information

Reporting Summary

Supplementary Information

## Data Availability

The datasets generated during and/or analyzed during the current study are available from the corresponding author on reasonable request.
