# Supplementary material for: Engineering large, anatomically shaped osteochondral constructs with robust interfacial shear properties
Source: NPJ Regen Med. 2021 Aug 6;6:42. doi: 10.1038/s41536-021-00152-0 (PMC8346478; doi:10.1038/s41536-021-00152-0)
Supplement: Supplementary file 1 — Reporting Summary [file 41536_2021_152_MOESM1_ESM.pdf]

nature research

Corresponding author(s): Kyriacos A. Athanasiou

Last updated by author(s): Jan 4, 2021

Reporting Summary

Nature Research wishes to improve the reproducibility of the work that we publish. This form provides structure for consistency and transparency in reporting. For further information on Nature Research policies, see our Editorial Policies and the Editorial Policy Checklist.

Statistics

For all statistical analyses, confirm that the following items are present in the figure legend, table legend, main text, or Methods section.

n/a	Confirmed	
☐	☒	The exact sample size (*n*) for each experimental group/condition, given as a discrete number and unit of measurement
☐	☒	A statement on whether measurements were taken from distinct samples or whether the same sample was measured repeatedly
☐	☒	The statistical test(s) used AND whether they are one- or two-sided<br>*Only common tests should be described solely by name; describe more complex techniques in the Methods section.*
☒	☐	A description of all covariates tested
☐	☒	A description of any assumptions or corrections, such as tests of normality and adjustment for multiple comparisons
☐	☒	A full description of the statistical parameters including central tendency (e.g. means) or other basic estimates (e.g. regression coefficient) AND variation (e.g. standard deviation) or associated estimates of uncertainty (e.g. confidence intervals)
☐	☒	For null hypothesis testing, the test statistic (e.g. *F*, *t*, *r*) with confidence intervals, effect sizes, degrees of freedom and *P* value noted<br>*Give P values as exact values whenever suitable.*
☒	☐	For Bayesian analysis, information on the choice of priors and Markov chain Monte Carlo settings
☐	☒	For hierarchical and complex designs, identification of the appropriate level for tests and full reporting of outcomes
☒	☐	Estimates of effect sizes (e.g. Cohen's *d*, Pearson's *r*), indicating how they were calculated

Our web collection on statistics for biologists contains articles on many of the points above.

Software and code

Policy information about availability of computer code

Data collection	ImageJ (NIH), TestBuilder V2 (Test Resources), Excel (Microsoft), LabView 5.1 (National Instruments),
Data analysis	JMP Pro 14 (SAS), Meshmixer (Autodesk), Inventor (Autodesk), Matlab 2019b (Mathworks), Prism 7 (Graphpad),

For manuscripts utilizing custom algorithms or software that are central to the research but not yet described in published literature, software must be made available to editors and reviewers. We strongly encourage code deposition in a community repository (e.g. GitHub). See the Nature Research guidelines for submitting code & software for further information.

Data

Policy information about availability of data

All manuscripts must include a data availability statement. This statement should provide the following information, where applicable:

- Accession codes, unique identifiers, or web links for publicly available datasets
- A list of figures that have associated raw data
- A description of any restrictions on data availability

The datasets generated during and/or analyzed during the current study are available from the corresponding author on reasonable request.

Field-specific reporting

Please select the one below that is the best fit for your research. If you are not sure, read the appropriate sections before making your selection.

☒ Life sciences ☐ Behavioural & social sciences ☐ Ecological, evolutionary & environmental sciences

For a reference copy of the document with all sections, see nature.com/documents/nr-reporting-summary-flat.pdf

Life sciences study design

All studies must disclose on these points even when the disclosure is negative.

Sample size	Sample size was determined to be n=8 per group based on a power analysis using pilot study data (means and SD) with thresholds of power=0.8 and alpha=0.05 using JMP Pro 14 (SAS).
Data exclusions	Statistical outliers were determined by using a ROUT outlier test in Prism 7 (GraphPad) and were removed before further statistical analysis.
Replication	Reproducibility of the experimental findings was achieved by repeating the experimental design in three times. All attempts at replication were successful.
Randomization	Samples were randomly assigned to each study group via simple sample randomization.
Blinding	Blinding was achieved by having one person randomize the order in which samples were tested; this person did not inform the person performing tests as to the sample identity. All analysis that could not be blinded was highly standardized to eliminate bias.

Reporting for specific materials, systems and methods

We require information from authors about some types of materials, experimental systems and methods used in many studies. Here, indicate whether each material, system or method listed is relevant to your study. If you are not sure if a list item applies to your research, read the appropriate section before selecting a response.

Materials & experimental systems

n/a	Involved in the study	
☐	☒	Antibodies
☒	☐	Eukaryotic cell lines
☒	☐	Palaeontology and archaeology
☒	☐	Animals and other organisms
☒	☐	Human research participants
☒	☐	Clinical data
☒	☐	Dual use research of concern

Methods

n/a	Involved in the study	
☒	☐	ChIP-seq
☒	☐	Flow cytometry
☒	☐	MRI-based neuroimaging

Antibodies

Antibodies used	ab34710, ab34712 (all from Abcam)
Validation	From the manufacturer (Abcam): "We use a variety of methods, including staining multi-normal human tissue microarrays (TMAs), multi-tumor human TMAs, and rat or mouse TMAs during antibody development. These high-throughput arrays allow us to check many tissues at the same time, providing uniformly as all tissues are exposed to the exact same conditions."<br><br>Both antibodies were also internally validated before use in this study via FFPE IHC on native sheep tissue that is well-known to be rich in collagen II and devoid of collagen I (articular cartilage) and on native tissue that is well-known to be rich in collagen I and devoid of collagen II (bone).
