# Supplementary material for: Engineering large, anatomically shaped osteochondral constructs with robust interfacial shear properties
Source: NPJ Regen Med. 2021 Aug 6;6:42. doi: 10.1038/s41536-021-00152-0 (PMC8346478; doi:10.1038/s41536-021-00152-0)
Supplement: Supplementary file 2 — Supplementary Information [file 41536_2021_152_MOESM2_ESM.pdf]

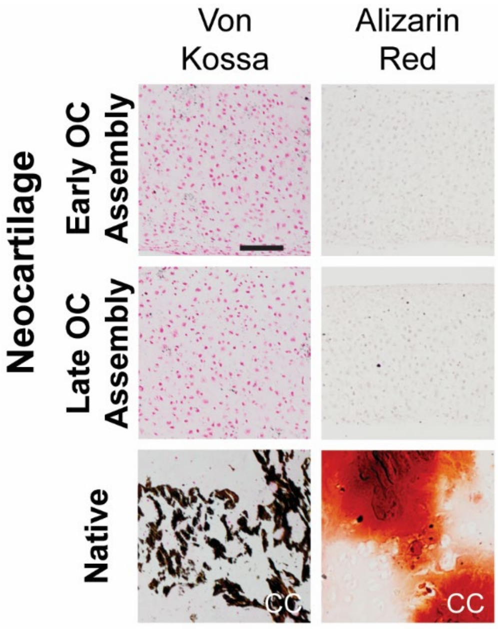

Supplementary Figure 1: Von Kossa and alizarin red staining. Neocartilage removed from the ceramics in both OC assembly groups did not stain with von Kossa or alizarin red. Native ovine costal cartilage (CC) is shown as control tissue. Scale bar represents 100 μm.

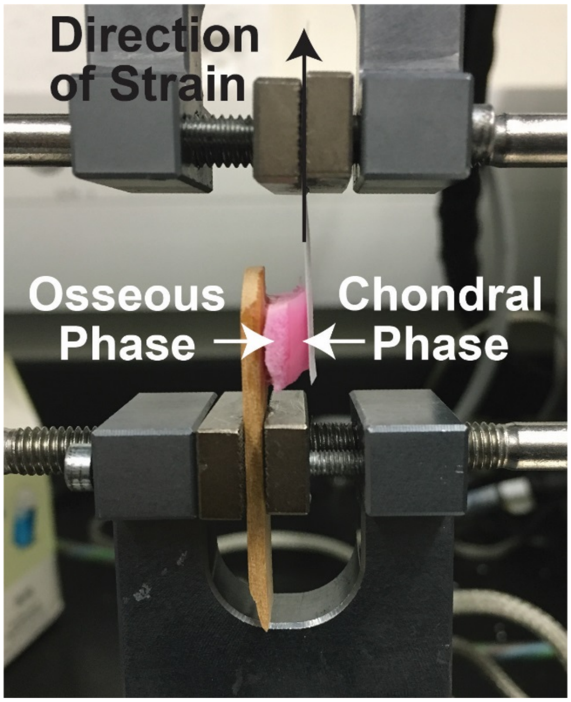

Supplementary Figure 2: Osteochondral lap shear testing set-up. The ceramic side of full-thickness osteochondral samples were glued to flat, wooden popsicle sticks to provide an immobile anchor. A paper tab was glued to the superficial surface of the neocartilage of the osteochondral samples. The wooden stick and paper tab were loaded into offset grips in the mechanical testing system such that the neocartilage-ceramic interface was parallel to the direction of strain. The sample was pulled apart at a rate of 1 mm/min until failure.
